# Genome-Wide Identification of the Cytochrome P450 Superfamily Genes and Targeted Editing of *BnCYP704B1* Confers Male Sterility in Rapeseed

**DOI:** 10.3390/plants12020365

**Published:** 2023-01-12

**Authors:** Zhilai Wang, Yanfeng Zhang, Min Song, Xiuhua Tang, Shuhua Huang, Bin Linhu, Ping Jin, Weike Guo, Fang Li, Liwen Xing, Ran An, Xiaona Zhou, Wenfang Hao, Jianxin Mu, Changgen Xie

**Affiliations:** 1 State Key Laboratory of Crop Stress Biology for Arid Areas and College of Life Sciences, Northwest A&F University, Yangling 712100, China; 2 Hybrid Rapeseed Research Centre of Shaanxi Province, Yangling 712100, China; 3 College of Agronomy, Northwest A&F University, Yangling 712100, China

**Keywords:** cytochrome P450, genic male sterility, *MS26*, CRISPR/Cas9, genome engineering

## Abstract

The cytochrome P450 (CYP450) monooxygenase superfamily, which is involved in the biosynthesis pathways of many primary and secondary metabolites, plays prominent roles in plant growth and development. However, systemic information about CYP450s in *Brassica napus* (BnCYP450) was previously undiscovered and their biological significance are far from understood. Members of clan 86 CYP450s, such as CYP704Bs, are essential for the formation of pollen exine in plant male reproduction, and the targeted mutagenesis of *CYP704B* genes has been used to create new male sterile lines in many crops. In the present study, a total of 687 *BnCYP450* genes were identified in *Brassica napus* cultivar “Zhongshuang 11” (ZS11), which has nearly 2.8-fold as many *CYP450* members as in *Arabidopsis thaliana*. It is rationally estimated since *Brassica napus* is a tetraploid oil plant with a larger genome compared with *Arabidopsis thaliana*. The *BnCYP450* genes were divided into 47 subfamilies and clustered into nine clans. Phylogenetic relationship analysis reveals that *CYP86* clan consists of four subfamilies and 109 *BnCYP450s*. Members of *CYP86* clan genes display specific expression profiles in different tissues and in response to ABA and abiotic stresses. Two *BnCYP450s* within the *CYP704* subfamily from *CYP86* clan, *BnCYP704B1a* and *BnCYP704B1b*, display high similarity to *MS26* (*Male Sterility 26*, also known as *CYP704B1*). These two *BnCYP704B1* genes were specifically expressed in young buds. We then simultaneously knocked-out these two *BnCYP704B1* genes through a clustered regularly interspaced short palindromic repeats/CRISPR-associated protein 9 (CRISPR/Cas9) genome engineering system. The edited plants displayed a pollenless, sterile phenotype in mature anthers, suggesting that we successfully reproduced genic male sterility (GMS, also known as nuclear male sterility) lines in *Brassica napus*. This study provides a systemic view of *BnCYP450s* and offers a strategy to facilitate the commercial utility of the CRISPR/Cas9 system for the rapid generation of GMS in rapeseed via knocking-out *GMS* controlling genes.

## 1. Introduction

Rapeseed (*Brassica napus* L., also known as canola, genome AACC, 2n = 4x = 38) is one of the most important vegetable oil crops across the world and is an allotetraploid that developed through doubling of chromosomes after the hybridization between *Brassica rapa* (genome AA, 2n = 20) and *Brassica oleracea* (genome CC, 2n = 18) [1,2]. The widespread adoption of hybrid vigor has contributed significantly to rapid and widespread increases in rapeseed production over the past few decades. Since then, the use of several pollination control systems has been proposed to promote heterosis in rapeseed, including cytoplasmic male sterility (CMS), genic male sterility (GMS), self-incompatibility, and chemical-induced male sterility (CIMS) [3]. Compared with other approaches, the major advantage of GMS is that it brings about nearly complete male sterility to a hybrid breeding program.

There are several GMS control loci used in hybrid rapeseed production, such as the S45AB, 117AB, Oro, 7365AB, 9012AB, Rs1046AB, Yi3A, and 609A systems [4,5,6,7,8]. In some of these systems, the genes controlling the male sterility have been determined. For instance, the male sterility of S45A and 117A is controlled by two duplicate genes, *BnMs1* and *BnMs2* [7]. The male sterile phenotypes of 7365A and 9012A are coupled with the loss of the *BnMs3* gene [5,6,8]. However, the main obstacle to using GMS remains the cross-breeding of the male sterility into new rapeseed varieties and there are only a few GMS lines available for conventional genetic manipulation. 

Recently, targeted mutagenesis of *GMS* control genes has been used to create new male sterile lines in many crops [9,10,11,12,13,14,15,16,17,18]. One of these *GMS* genes is *ZmMS26* (also known as *ZmCYP704B1*), which is a cytochrome *P450-like* gene first identified in maize [15,16,19]. *ZmMS26* encodes long-chain fatty acid omega-hydroxylase required for sporopollenin biosynthesis and essential for pollen exine formation [11,15]. Since then, protein sequences with high homology to the ZmMS26 were identified, and targeted mutagenesis was performed to create new male sterile germplasms in many plants, such as maize, rice, sorghum, and wheat [11,14,15,16,20]. However, the rapid deployment of genic male sterile lines of rapeseed at the commercial breeding level derived from artificial manipulation remains controversial.

The cytochrome P450 (CYP450) monooxygenase superfamily is one of the largest enzymatic protein families of heme-thiolate proteins [21,22]. The CYP450s catalyze a great number of NADPH- and/or O_2_-dependent oxygenation/hydroxylation reactions in many organisms [21,23]. The *CYP450* genes are widely identified in most organisms, including bacteria, plants, animals, and humans [21,22,23,24,25,26,27,28]. In plants, the nomenclature of *CYP450* genes is generally classified into two groups: A type and non-A type [21,22]. The A type *CYP450s* are specific to plants; the non-A type is non-plant-specific *CYP450s* which display high sequence similarity to animals and fungal *CYP450s* [22,23]. According to the current evolutionary relationship, plant *CYP450* subfamilies are grouped into different clans including five single-subfamily clans (clan 51, clan 710, clan 711, clan 74 and clan 97) and four multiple-subfamily clans (clan 72, clan 85, clan 86 and clan 71) [21,22,24]. The clan 71 was categorized into A type *CYP450s*, and the other eight clans were categorized into non-A type *CYP450s* [21,22,23]. 

A great number of *CYP450* genes have crucial roles in the biosynthesis of metabolites with distinct biological significance [21,25]. Plenty of the clan 73, clan 84, and clan 98 *CYP450* genes are involved in the phenylpropanoid biosynthetic pathway, results in producing a mass of phenol related compounds, i.e., flavonoids, suberin, lignin, and polyphenols [21,25]. Members of the *CYP703*, *CYP704*, and *CYP86* subfamilies are responsible for the biosynthesis of pollen exine and anther cutin, which are known as fatty acid hydroxylase [7,29,30,31,32]. The *CYP701A* and *CYP88A* genes are necessary for gibberellic acid (GA) biosynthesis, which encode ent-kaurene oxidase and ent-kaurenoic acid oxidase respectively [24,33,34]. The CYP707A is involved in abscisic acid (ABA) 8′-hydroxylation, which play an important role in ABA catabolism [35,36]. The CYP450 enzymes of the CYP74 subfamily are essential for jasmonate (JA) synthesis, which function as allene oxide synthase (AOS) [24,37]. Members of the CYP90, CYP72, CYP92, CYP85, CYP734, and CYP724 subfamilies are necessary for brassinosteroid (BR) biosynthesis [21,38]. Besides, CYP450s also act in fighting against abiotic and biotic stresses [21].

In the current study, we systematically identified and analyzed the *BnCYP450* genes in the rapeseed genome. In addition, we report the introduction of GMS into rapeseed by simultaneously knocking-out two anther-specific *ZmMS26* homologs through gene editing using the CRISPR (clustered regularly interspaced short palindromic repeats)/Cas9 (CRISPR-associated protein 9) system.

## 2. Results 

### 2.1. Genome-Wide Identification of BnCYP450s

There are 687 putative BnCYP450s with conserved P450 domains isolated from the genome of *Brassica napus* cultivar “Zhongshuang 11” (ZS11), which is around 2.8 folds of AtCYP450s. This is not surprising since *Brassica napus* is tetraploid and Arabidopsis is diploid with a very small genome size. We found 318 and 367 *BnCYP450* genes in the A and C subgenome respectively (Table 1). The *BnCYP450s* were renamed in terms of their sequences’ similarity to *AtCYP450s* (Table S1). The protein length of BnCYP450s was between 80 and 1971 amino acids (aa) with predicted molecular weight (Mw) in the range of 8.67–224 kDa. The theoretical isoelectric points (pI) of BnCYP450s ranged from 4.1 to 11.52 (Table S1). The pI of 547 BnCYP450s (79.6%) showed values greater than 7. Other characteristics of BnCYP450s, such as gene position and accession number, are presented in Table S1.

### 2.2. Classification and Phylogenetic Analyses of BnCYP450 Proteins

We divided BnCYP450 proteins into 46 subfamilies according to the P450 nomenclature (Table S1). To explore the evolutionary relationships between AtCYP450s and BnCYP450s, an unrooted NJ tree with the 251 AtCYP450s and 687 BnCYP450s was constructed (Figure S1). Based on it, BnCYP450s were also categorized into nine clans. However, there were no members identified in CYP716 subfamily. The *BnCYP450s* were also classified into non-A type and A-type, which is consistent to previous evolutionary analysis [23]. The non-A type BnCYP450s consisted of eight clans with 27 subfamilies containing 275 members. The A-type BnCYP450s were all grouped into CYP71 clan with 19 subfamilies comprising 412 members (Table 1). The CYP721, CYP734, CYP718, CYP720, CYP701, CYP75, and CYP93 subfamilies all had only two members, while the largest subfamily CYP71 harbored 138 members.

### 2.3. Gene Duplication and Collinearity Analysis of the BnCYP450 Genes

**Figure 1:**
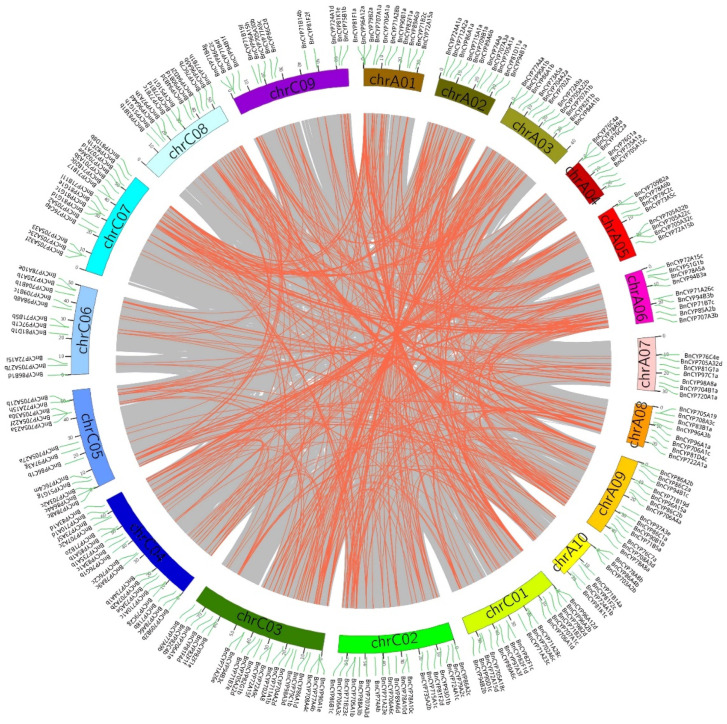
The synteny analysis of *BnCYP450* genes in *Brassica napus*. The gray lines display all synteny blocks, and the red lines indicate the segmental duplicate gene pairs in *Brassica napus* Cultivar ZS11 genome respectively.

To study the putative tandem and segmental duplication events, we determined the chromosomal distribution of *BnCYP450* family members. In total, the isolated 687 *BnCYP450* genes, except *BnCYP81F4a* and *BnCYP94B2a*, were widely distributed on 19 chromosomes (Table S1). *BnCYP81F4a* and *BnCYP94B2a* were anchored on HiCscaffold2150 and HiCscaffold2213 respectively. The distributing density of *BnCYP450s* on chromosomes was extremely uneven. A relatively high density was observed on chromosomes A09, C03 and C07 (more than 50 genes) while a relatively low density on chromosomes A07, A10 and C06 (fewer than 20 genes). We identified a total of 32 tandem duplication events and 608 segmental duplications in the *Brassica napus* genome according to the BLAST and MCScanX results (Figure 1 and Tables S2 and S3). Moreover, only nine segmental duplication events were observed within the same chromosome and five of them were observed on chromosomes C04, whereas 599 segmental duplications were detected across chromosomes (Table S3). Further analysis revealed that there are 88 duplication events located on the AA subgenome and 91 events on the CC subgenome, whereas there are 429 events occurred between AA and CC subgenomes (Tables S4 and S5). In terms of the U triangle, allopolyploidization may have key roles in the expansion of the *BnCYP450s* in *Brassica napus.* To ascertain the evolution of the *BnCYP450* genes, we determined the synteny between *Brassica napus* and *Arabidopsis thaliana* at the whole genome level. A total of 458 collinear gene pairs were observed between the two genomes (Figure S2 and Table S6). Most (113 out of 142) *AtCYP450s* genes have multiple orthologous genes in *Brassica napus*. For instance, AtCYP706A4 has 11 collinear *BnCYP450* genes. However, there are 29 *AtCYP450* genes having only one collinear *BnCYP450* gene (Table S6).

### 2.4. Gene Structure Analyses of the Clan 86 BnCYP450 Genes

**Figure 2:**
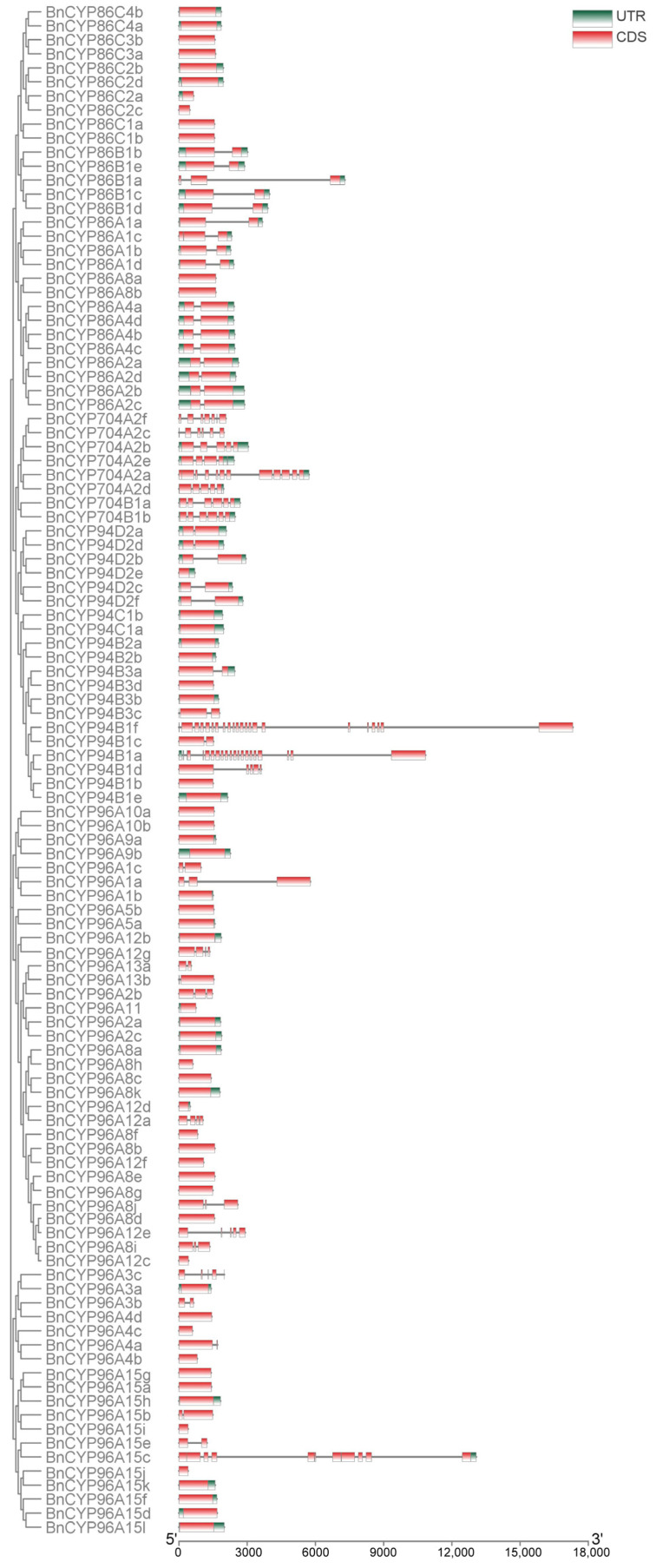
Schematic exon/intron structures of the clan 86 *BnCYP450* genes. The red boxes represent exons and black lines represent introns. The UTR region of are indicated in green boxes. The length of CDS can be estimated by the scale at the bottom.

As above mentioned, members of clan 86 CYP450s, such as CYP704Bs, are essential for the formation of pollen exine in plant male reproduction and targeted mutagenesis of *CYP704B* genes has been used to create new male sterile lines in many crops [11,14,16,30]. Therefore, we are focused on analysis of clan 86 CYP450s. The overall exon/intron profile is an index to understand the phylogenetic relationships within a particular gene family [39]. So, we first determined the intron and exon organization of the clan 86 *BnCYP450* genes. The majority of *CYP96* subfamily genes (29 out of 52) contains no intron. Most members of the *CYP86* (12 out of 29) and *CYP94* (9 out of 20) subfamilies also have intronless genes (Figure 2). All members of the *CYP704* subfamily have at least three introns. There are two genes from *CYP94* subfamily (*BnCYP94B1a* and *BnCYP94B1f*) and one gene from *CYP96* subfamily (*BnCYP96A15c*) having at most 21 introns (Figure 2). In short, these gene architectures within a subfamily may also be attributed to the high quality of ‘ZS11’ genome assembly after comparing with the ‘Darmor-bzh’ genome assembly as previously described [1]. 

### 2.5. Tissue-Specific Transcript Accumulation Patterns of the Clan 86 BnCYP450 Genes

**Figure 3:**
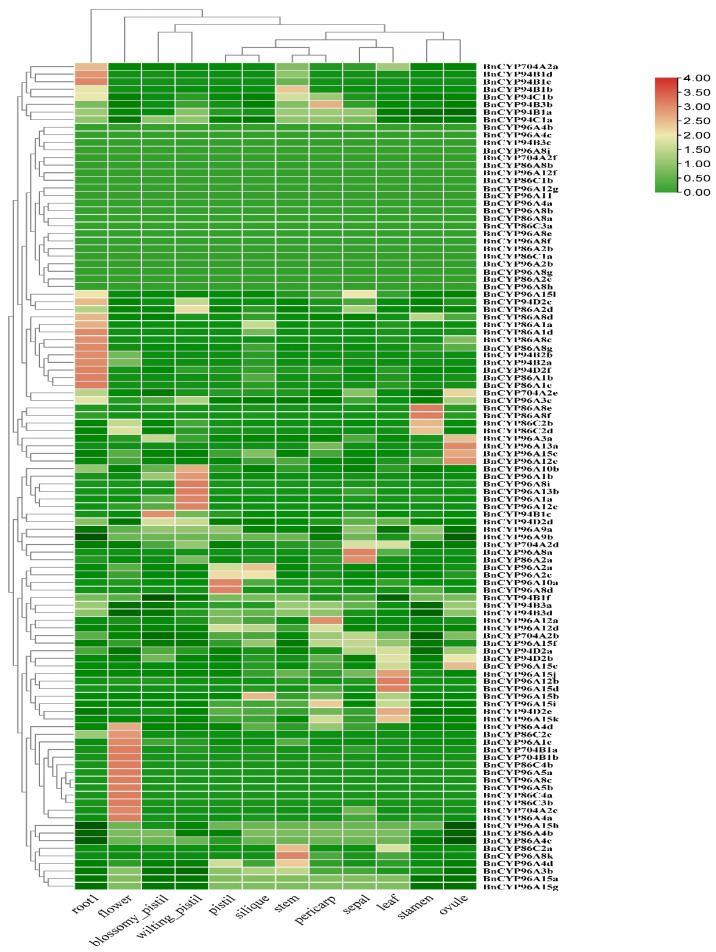
Expression profiles of the clan 86 *BnCYP450* genes in different tissues. The expression level is equal to the mean values and transforms log_2_ values for normalization. The color scale represents relative expression levels from low (green colored) to high (red colored).

In order to determine the transcript abundance of the clan 86 *BnCYP450* genes, we investigated their expression profiles in 12 different tissues (stem, sepal, pistil, stamen, ovule, pericarp, blossomy pistil, wilting pistil, root, flower, leaf and silique) using public available RNA-seq data of ZS11 as above mentioned [40,41]. Eighty-seven genes were observed to be expressed in at least one tissue, while 22 genes were not observed to be expressed in any of the analyzed tissues (Figure 3 and Table S7). Moreover, three genes were specifically expressed in one tissue, whereas 24 genes were expressed in all of the analyzed tissues (Figure 3 and Table S7). There are 13 genes highly expressed in flower. These results suggest that some members of the clan 86 *BnCYP450s* may have distinct roles in cell differentiation and tissue development, and the flower expressed members may play conserved roles in control of fertility.

### 2.6. Expression Profiles of the Clan 86 BnCYP450 Genes in Response to Abiotic Stresses

**Figure 4:**
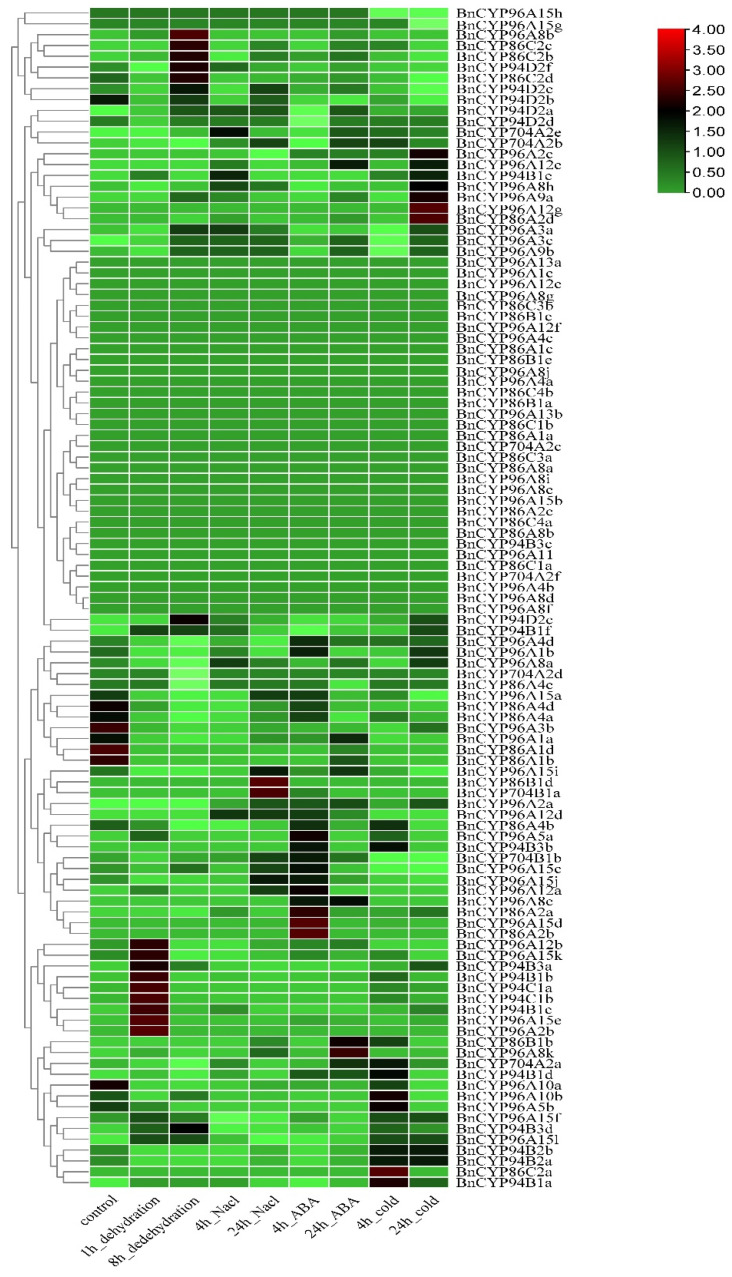
Expression profiles of the clan 86 *BnCYP450* genes under different stress conditions. The expression level is equal to the mean values and transforms log_2_ values for normalization. The color scale represents relative expression levels from low (green colored) to high (red colored).

To examine the transcript abundance of the clan 86 *BnCYP450* genes in response to abiotic stresses, we investigated their expression profiles under dehydration, NaCl, ABA, and cold conditions with publicly available RNA-seq data of ZS11 as above mentioned [40,41]. Sixty-seven genes were observed to be induced in response to at least one analyzed treatment, while nine genes were observed to be repressed in response to at least one analyzed treatment (Figure 4 and Table S8). There were 33 genes that could not be detected in response to any of the analyzed treatments. Moreover, there are some genes significantly induced by certain treatments, such as *BnCYP94B3a* and *BnCYP96A15l* by dehydration and cold or *BnCYP94B1d* by ABA. Consistent with a previous study [30], the expression of some *BnCYP704* genes, such as *BnCYP704B1b*, *BnCYP704A2e*, *BnCYP704A2b*, *BnCYP704A2d*, *BnCYP704A2a*, and *BnCYP704B1a*, are affected by abiotic stresses (Figure 4 and Table S8).

### 2.7. The Brassica Napus Genome Encodes Two Putative ZmMS26-Like Genes

**Figure 5:**
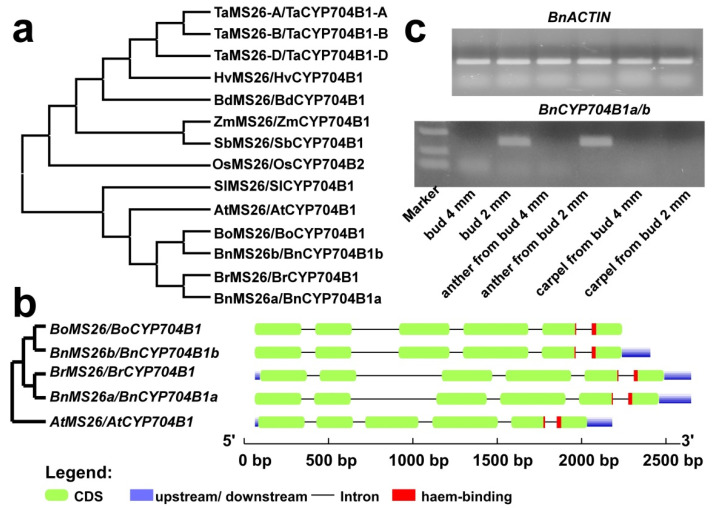
Phylogenetic analysis of MS26 orthologs and anther-specific expression of *BnCYP704B1a* and *BnCYP704B1b.* (**a**) Phylogenetic analysis of MS26 orthologs. The protein sequences of MS26 orthologs were aligned by the MUSCLE tool; the maximum likelihood tree was generated using MEGA 6.0. (**b**) Genetic architecture of *BoMS26*, *BnCYP704B1b*, *BrMS26*, *BnCYP704B1a* and *AtMS26*. (**c**) RT-PCR analysis of *BnCYP704B1a* and *BnCYP704B1b* in buds, anther and carpel released from buds using *BnACTIN* as control. The exon/intron distribution of selected *MS26* genes was determined using the online Gene Structure Display Server 2.0. The CDS (corresponding to exons) is represented by green boxes. The introns are indicated as black lines. The haem-binding motif region on the genomic sequence is denoted by red box.

As displayed in Figure 5a and Table S1, there are two orthologous sequences for AtMS26 found in the rapeseed genome, *BnCYP704B1a* (ZS11A07G034230) and *BnCYP704B1b* (ZS11C06G043970), both of which contained six exons and five introns (Figure 5b). To remain consistent with previous MS26 nomenclature, we renamed *BnCYP704B1a* as *BnMS26a* and *BnCYP704B1b* as *BnMS26b*, respectively. Both *BnCYP704B1a* and *BnCYP704B1b* encode predicted proteins with 519 amino acids, estimated molecular masses of 59.8 kDa, and predicted pI of 8.58. Comparison of the predicted amino acid sequences and coding sequences of *BnCYP704B1a* and *BnCYP704B1b* revealed that they are highly similar, displaying about 98% amino acid identity (Figure S3) and 97% nucleotide identity (Figure S4). All of these indicate that *BnCYP704B1a* and *BnCYP704B1b* are segmental duplicating genes in rapeseed, and they originated from different progenitor species according to the U triangle [2]. In other words, *BnCYP704B1a* originated from *Brassica rapa* and *BnCYP704B1b* originated from *Brassica oleracea*. Phylogenetic analysis shows that ZmMS26 homologs in monocots (rice, maize, sorghum, Brachypodium, tomato and wheat) and dicot (the Brassicaceae species Arabidopsis, cabbage and rapeseed) are divided into two subfamilies (Figure 5a). 

### 2.8. BnCYP704B1a and BnCYP704B1b Are Mainly Expressed in Young Anther 

The expression of the *ZmMS26* gene is limited to developing anthers [11,15,16], which is consistent with its role in pollen development. Moreover, the *ZmMS26* orthologs in rice, sorghum, and wheat also confer male sterility when targeted by mutagenesis, strongly supporting the conserved function in anthers [11,15,16]. To examine the expression patterns of the *BnCYP704B1a* and *BnCYP704B1b* genes, we first compared their expression levels in different tissues by analyzing available RNA-seq data from rapeseed [42]. The expression of *BnCYP704B1a* and *BnCYP704B1b* was only significantly detected in young buds (around 2 mm in diameter, Figure S4). To gain an insight into whether they also have a conserved anther-expressed pattern, we determined the expression levels of *BnCYP704B1a* and *BnCYP704B1b* in different flower tissues using RT-PCR. Concerning their high sequence identity, we used degenerate primers to detect their combined expression. Consistent with the transcriptome data, the expression of *BnCYP704B1a* and *BnCYP704B1b* could be only detected in young buds (Figure 5c). In flower parts isolated from young buds, the expression of *BnCYP704B1a* and *BnCYP704B1b* was only detected in anther, but not in carpel (Figure 5b and Figure S5), suggesting that they may also have a role at the very early stage of pollen development. 

### 2.9. BnCYP704B1a and BnCYP704B1b Simultaneous Knockout Mutants Are Male Sterile

**Figure 6:**
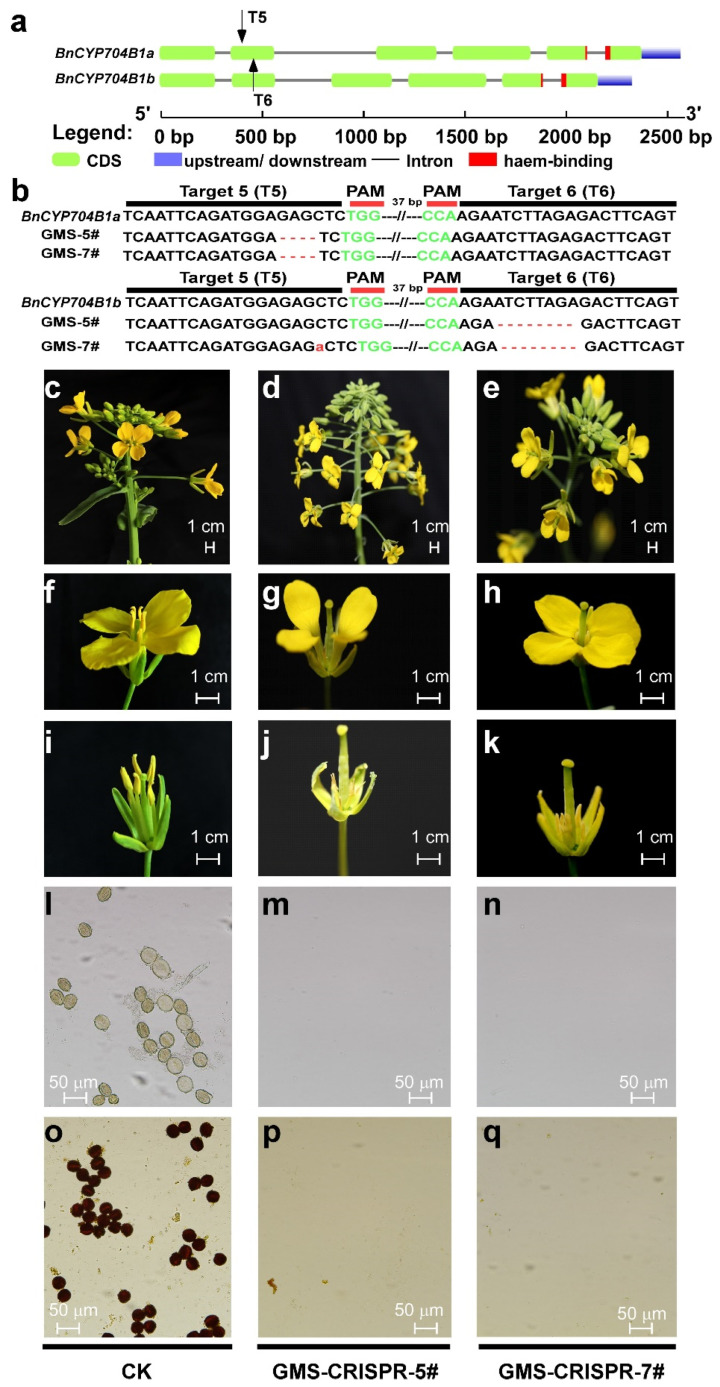
Male sterility phenotypes of CRISPR/Cas9-edited GMS lines. (**a**) Gene structures of *BnCYP704B1a* and *BnCYP704B1b* and the corresponding CRISPR/Cas9 guide RNA target sites, marked by black arrows. (**b**) Mutated genotypes of *BnCYP704B1a* and *BnCYP704B1b* after CRISPR/Cas9 editing. The target sites sequences are marked in *BnCYP704B1a* and *BnCYP704B1b* sequences, with DEFINE (PAM) highlighted in green. (**c**–**e**) inflorescence; (**f**–**h**) flower; (**i**–**k**) flower without petal; (**l**–**q**) pollen fertility in CK (represents the recipient line K407) and CRISPR/Cas9-edited plants; (**o**–**q**) the pollen was stained with I_2_—KI solution. No detectable pollen was observed from CRISPR/Cas9-edited GMS plants. (**c**–**k**), scale bars, 1 cm; (**l**–**q**), scale bars, 50 μm.

To determine whether the *BnCYP704B1a* and *BnCYP704B1b* could be edited by CRISPR/Cas9 system, we designed six sgRNA (Target-1~Target-6) to simultaneously target the first or the second exon (Figure 6a, Table S9). The six sgRNAs were divided into three groups and inserted into pHSE401 expression vectors as two gRNA expression cassettes [43]. The resulting three recombinant plasmids were transformed into *Agrobacterium tumefaciens* and co-cultured with the hypocotyls of rapeseed. As previously reported, CRISPR/Cas9-induced editing events could take place in the callus cells or in the regenerated shoots derivate from transformed hypocotyl before regeneration in rapeseed [44]. As expected, homozygous mutants with gRNA-directed mutation were acquired in the regenerated shoots (Figure S6). The editing efficiency of each sgRNA expression cassette was measured in the regenerated shoots. The sgRNA expression cassette harboring Target-1 and Target-2 was more likely to edit at both target sites (Figure S6). The sgRNA expression cassette harboring Target-5 and Target-6 was prone to edit at target site 5 (Figure S6). No editing event was observed at target sites 3 and 4 (Figure S6). Moreover, editing events with deletions were frequently observed in *BnCYP704B1a* and *BnCYP704B1b* in all of the regenerated shoots (Figure S6). We obtained two T_2_ plants harboring mutations in both *BnCYP704B1a* and *BnCYP704B1b* from the edited shoots (Figure 6b). All of these lines exhibited a pollenless, sterile phenotype in mature anthers (Figure 6). Sequencing results confirmed that all of these lines harbored the editing events at the targeted sites observed in the regenerated shoots (Figure 6b). To investigate whether the deletions generated by the CRISPR/Cas9 system could be transmitted to the next generation, they were cross-pollinated with the recipient line K407. The fertility of the corresponding F_1_ and F_2_ progenies were determined. All F_1_ progenies developed normal pollen grain in the mature anthers and produced seeds via self-fertilization. Around a quarter of the F_2_ progeny plants displayed the pollenless, sterile phenotype, indicating that *BnCYP704B1a* and *BnCYP704B1b* are two duplicate, recessive genes. Sequencing results also confirmed that the observed editing events were able to be transmitted to the next generations. Furthermore, the sterile edited *BnCYP704B1a* and *BnCYP704B1b* plants were able to set seed via artificial pollination with pollen from the recipient line K407, indicating normal female organ development and fertilization potential.

To examine whether any off-target event occurs, we determined the potential off-target sites for *BnCYP704B1a* and *BnCYP704B1b*, respectively. After sequencing the amplified fragments of those potential off-target sites, no genome editing was observed in any examined samples (Table S10). These results indicated that the two sgRNA expression cassettes harboring Target-1 and Target-2 or Target-5 and Target-6 had the highest editing efficiency and an undetectable off-target effect. 

## 3. Discussion 

In the present study, a total of 687 *BnCYP450* genes were identified from *Brassica napus* cultivar ZS11, which is assigned into 22 subfamilies and clustered into nine clans (Table 1). The number of *BnCYP450s* was greater than those of Arabidopsis (251), *Brassica rapa* (354), *Brassica oleracea* (343), rice (355), apple (348), tomato (457), cotton (461), model legume (346), grape (236), and fewer than in wheat (1700) [22,23,24,25,27,28]. Some subfamilies and clans showed deep phylogenetic relationships. For example, the CYP720 subfamily was inside the CYP90 subfamily on the CYP85 clan branch. The CYP704 subfamily was separated into two clades by the CYP94 and CYP86 subfamily on the CYP86 clan branch. The CYP89 subfamily was clustered inside the CYP77 subfamily on the CYP71 clan branch. The CYP76 subfamily from the CYP71 clan was separated into two clades by the CYP72 clan on the phylogenetic tree (Figure S1). Based on the above analysis, we speculated that the CYP450 superfamily has a significant degree of evolutionary extension in *Brassica napus*. Moreover, the large range of Mw and pI of BnCYP450s may determine their functional diversity in distinct biosynthetic pathways.

It is conceivable that gene duplication represents a major force to gene expansion [21,40,45]. Compared with those of *AtCYP450s* (251), *BrCYP450s* (354), and *BoCYP450s* (343), the number of *BnCYP450s* (687) was significantly expanded in *Brassica napus*, which may be attributed to the genome polyploidy event in terms of the U’ triangle [1,2,46]. Indeed, there were 32 tandem duplication events and 608 segmental duplications observed across AA and CC subgenomes (Table S3). Moreover, there were 88 and 91 duplication events observed within AA or CC subgenomes, respectively (Tables S4 and S5). So, tetraploid, segmental, and tandem duplication are the main types of gene duplication events taking place in the *BnCYP450s* superfamily. Under evolutionary pressures, duplication events have extended the gene family members and mutations in the upstream regions can alter the expression patterns of new members [47,48].

As mentioned above, some members of the CYP86 clan (CYP704, CYP86 and CYP94 subfamilies) are recognized as fatty acid hydroxylases responsible for the biosynthesis of pollen exine and anther cutin [7,21,29,30,31,32]. It is tempting to speculate that gene expression patterns are clues to their biological significance [21,39,49]. The expression patterns of the *CYP86* clan *BnCYP450s* genes from 12 tissues and four types of abiotic stresses were determined. There were only a few of the *CYP86* clan *BnCYP450s* genes displayed tissue-specific expression (Figure 3 and Table S7), such as *BnCYP96A8c*, *BnCYP96A5b* and *BnCYP96A5a*. It is interesting that most of the *CYP86* clan *BnCYP450s* genes are induced by at least one analyzed treatment (Figure 4 and Table S8). Further RT-PCR analysis revealed that *BnCYP704B1a* and *BnCYP704B1b* are specifically expressed in young anther (Figure 5b), neither of which could be detected in any of the analyzed tissue or treatment (Figure 3 and Figure 4 and Tables S7 and S8). Concerning the importance of *BnCYP450* superfamily members, the expression patterns of the *CYP86* clan *BnCYP450s* examined in the present study may provide an important way to uncover the biological functions of other *BnCYP450s* in future.

Gene editing by the CRISPR/Cas9 system provides a rapid and specific way to manipulate the genetic material used in crop breeding. Since its first application in plants in 2013, targeted mutagenesis of interested genes has been used to create new germplasms in many crops [11,12,14,17]. One of these strategies is to target the mutagenesis of male-sterile control genes to create new male sterile lines in crops [9,11,12,13,14,18,20]. One of such male-sterile control genes is *ZmMS26* [7,11,16,20,29,31,32]. Targeted mutagenesis of orthologues of MS26 have created male sterile lines in maize, rice, wheat, sorghum, and bread wheat [11,16,20]. In the current work, gene editing directed the knock-out of two orthologues of *MS26* in *Brassica napus* is capable of generating a pure male sterile line (Figure 6). Moreover, editing events with deletions were frequently observed upon *BnCYP704B1a* and *BnCYP704B1b* in the obtained male sterile plants. This may be mainly attributed to the error-prone character of the two sgRNA expression cassettes, in which sgRNA directed deletion is observed to occur at high frequencies [43,50]. Target sequences with high editing and low off-target efficiencies will improve the breeding efficiency and reduce the breeding cost associated with the development of commercially applied genic male sterility lines. 

We have also established male sterile lines in tomato via CRISPR/Cas9 directed knock-out of tomato *CYP704B1* homolog [14]. As previously described, the conserved haem-binding loop in the C-terminal is essential for catalytic activity of haem-thiolate cytochrome P450s [11,16,20]. Targeted mutagenesis of the conserved haem domain of ZmMS26 (mainly located in exon 5 or exon 6) orthologs confers male sterility in monocots (such as maize, rice, sorghum, wheat) [11,16,20]. As mentioned above, our designed gRNAs were mainly located in exon 1 and exon 2 and conferred male sterility when simultaneously edited (Figure 6). Taken together, these results indicated that CRISPR/Cas9 directed simultaneously knock-out of *ZmMS26* orthologs could be used as a universal strategy to establish male sterile lines in allotetraploid plants (such as rapeseed and wheat). 

It is well-known that propagating a pure male sterile line is essential for commercial hybrid seed production [10]. The created male sterile line in the present study can serve as the foundation for applications in the two-line hybrid breeding of rapeseed. Taken together, our findings suggest that practical pure male sterile lines can be developed by the simultaneous, targeted knock-out of *BnCYP704B1a* and *BnCYP704B1b* using the CRISPR/Cas9 system, which is an important step towards capturing heterosis in commercial hybrid rapeseed production. Isolation of the inherited mutations in Cas9-free (also known as transgene clean) plants can ensure the stable transmission of the identified mutations to next generations. Moreover, the screening process for the male sterile line is time-consuming, laborious, and inefficient (50% fertile plants need to be removed before fertilizing). A long period of plant regeneration could be avoided with the selection of pure male sterile plants in advance, which are almost indistinguishable from classical male sterile lines during the molecular breeding period. Recently, a fluorescence-based seed sorting strategy has been adopted in the streamlined identification of male sterile lines in rice [10]. A convenient and efficient DsRed-based visual screening method has also been established in *Brassica napus* [51]. To further simplify the screening procedure for pure male sterile plants, a similar strategy also needs to be developed in hybrid rapeseed production in future. 

## 4. Materials and Methods

### 4.1. Identification of the Cytochrome P450 Gene Superfamily in Brassica napus

Protein sequences of the rapeseed cultivar ZS11 were obtained from National Genomics Data Center (NGDC, accession number PRJCA002883) [1]. The *cytochrome P450* genes of *Brassica napus* (*BnCYP450s*) were isolated from ZS11 through the HMM profile corresponding to the Pfam cytochrome P450 family PF00067 by running the HUMMER3.1 software of Linux version, with the threshold was e-value < e^−10^. A total of 251 AtCYP450 protein sequences download from the cytochrome P450 homepage (https://drnelson.uthsc.edu/, accessed on 11 November 2022) [22] were used as queries to perform a BLASTP search in the local protein database of ZS11. Then, the putative BnCYP450s were obtained via taking the intersection of the HUMMER and BLASTP methods. Finally, these proteins were submitted to the NCBI-CDD server (http://www.ncbi.nlm.nih.gov/Structure/cdd/wrpsb.cgi, accessed on 11 November 2022) and the SMART (Simple Modular Architecture Research Tool, http://smart.embl-heidelberg.de/, accessed on 11 November 2022) database to perform the cytochrome P450 domain predictions as described [40]. The theoretical molecular weight and isoelectric points of BnCYP450s were calculated by DNAstar as previously described [49].

### 4.2. Gene Duplication and Genomic Synteny of BnCYP450s

Gene duplication and genomic synteny of *BnCYP450s* were determined as previously described [40]. All of the BnCYP450 protein sequences were aligned using BLASTP with the threshold was e-value of e^−100^. Then, the duplication pattern of *BnCYP450* genes was determined through the MCScanX software with default parameters and divided into tandem and segmental duplication as previously described [40,52]. Similarly, all of the BnCYP450 and AtCYP450 protein sequences were aligned using BLASTP with the threshold e-value of e^−100^, and all putative syntenic blocks were mapped with JCVI software as described [40,46].

### 4.3. Phylogenetic and Gene Structure Analysis 

Protein sequences of the clan 86 BnCYP450 members were aligned with the FFT-NS-I method of the MAFFT software at first. The phylogenetic tree was constructed via FastTree software with the maximum likelihood method as previously described [40,53]. Then, the phylogenetic tree was visualized by Figtree software [39]. The overall intron/exon organization of the clan 86 *BnCYP450* genes were displayed based on GFF annotation files by TBtools software as previously described [40,54]. Several MS26 orthologs, namely AtMS26 (*Arabidopsis thaliana* MS26, At1g69500) [29], BoMS26 (*Brassica oleracea* MS26, Bo6g108940) [31], BrMS26 (*Brassica rapa* MS26, Bra004386), BnMS26a (*Brassica napus* MS26a), BnMS26b (*Brassica napus* MS26b), SlMS26 (*Solanum lycopersicum* MS26, XM_004228326.4) [14], OsMS26 (*Oryza sativa* MS26, XP_015629295.1), HvMS26 (*Hordeum vulgare* MS26, BAK08270.1), ZmMS26 (*Zea mays* MS26, NP_001130648.10) [11,15,20], SbMS26 (*Sorghum bicolor* MS26, XP_002465796.1), BdMS26 (*Brachypodium distachyon* MS26, XP_003558727.1), TaMS26-A (*Triticum aestivum* MS26-A, TraesCS4A03G0032700.1), TaMS26-B (*Triticum aestivum* MS26-B, TraesCS4B03G0752700.1), and TaMS26-D (*Triticum aestivum* MS26-D, TraesCS4D03G0673200.1) [16], were aligned using the MUSCLE tool, and the maximum likelihood trees were generated using MEGA 6.0. The online Gene Structure Display Server (GSDS2.0, http://gsds.cbi.pku.edu.cn/ accessed on 30 November 2022) was used to decipher the intron-exon distribution of *AtMS26*, *BoMS26*, *BrMS26*, *BnMS26a*, and *BnMS26b* genes as previously described [31].

### 4.4. Expression Profiles of the Clan 86 BnCYP450 Genes

RNA-seq data from different tissues and under abiotic stresses of ZS11 were downloaded from the NGDC (accession numbers PRJNA394926 and CA001775) [2]. All of these RNA-seq data were mapped to the reference genome of ZS11 with HISAT2 software [55]. Transcript abundance of the clan 86 *BnCYP450* genes was calculated by the TPM (Transcripts Per Million) values with FeatureCounts R package and a histogram was generated via TBtools software as previously described [54]. 

### 4.5. Determination of the Expression Profiles of BnCYP704B1a and BnCYP704B1b 

*Brassica napus* RNA-seq data derived from different tissues (http://yanglab.hzau.edu.cn/BnTIR, accessed on 11 November 2022) [42] was used to profile the expression levels of *BnCYP704B1a* and *BnCYP704B1b*. To examine the anther-specific expression of *BnCYP704B1a* and *BnCYP704B1b*, semi-quantitative RT-PCR was used. Briefly, a total RNA was extracted from young buds (around 2 mm or 4 mm in diameter), anther (isolated from young buds), and carpel (isolated from young buds) with TRIzol reagent (TaKaRa). The complementary DNA (cDNA) was obtained after reverse transcription with M-MLV Reverse transcriptase (Promega). Semi-quantitative RT-PCR was performed to assess the expression levels of *BnCYP704B1a* and *BnCYP704B1b* and was normalized to ZS11C02G003910, which is an *actin*-like gene in *Brassica napus*. 

### 4.6. Guide RNA Design and CRISPR/Cas9 Vector Construction

Guide RNAs targeting *BnCYP704B1a* and *BnCYP704B1b* were designed by CRISPR RGEN Tools (http://www.rgenome.net/cas-designer/, accessed on 11 November 2022), which provides both bulge-allowed RNA-guided Endonuclease (RGEN) targets via Cas-Designer [56] and potential off-targets within a 2-nt mismatch and optional 3-nt bulges via Cas-OFFinder [57]. It is advised to design at least two guide RNAs when intending to knock-out a single gene [43,56,57]. Since we were targeting two homologous genes with high sequence similarity, guide RNAs targeting either *BnCYP704B1a* or *BnCYP704B1b* were chosen to co-edit them. The CRISPR/Cas9 toolkit was set to design two guide RNA expression cassettes for genome editing in rapeseed as described previously [44]. Briefly, two guide RNAs were incorporated into two forward or two reverse primers, respectively, with the two forward or two reverse primers partially overlapping. The expression cassettes were amplified from pCBC-DT1T2 with these guide RNA-incorporated primers. Purified expression cassettes were digested with *Bsa*I and inserted into pHSE401 via T4 DNA ligase (TaKaRa) reactions. The oligos used to construct the CRISPR/Cas9 vector for *BnCYP704B1a* and *BnCYP704B1b* (pHSE401-*BnCYP704B1*) are listed in Table S9. 

### 4.7. Plant Material and Plant Transformation

Seeds of the winter rapeseed selfing line K407 were obtained from Hybrid Rapeseed Research Centre of Shaanxi Province, China. K407 shows high transformation efficiency (from our preliminary data, not shown). Rapeseed hypocotyl transformation followed the protocol as previously described [44,51], with modifications to *Agrobacterium tumefaciens* depletion and hygromycin selection. Briefly, seeds were surface sterilized and germinated in the dark. After 5–6 days, the etiolated hypocotyls were cut into 0.8–1-cm segments and co-cultivated with *Agrobacterium tumefaciens* harboring the pHSE401-*BnCYP704B1* construct for 2 days in a solid M1 medium. Following co-cultivation, the hypocotyl explants were placed on M2 medium with 300 μg/mL Timentin and 10 μg/mL hygromycin to remove *Agrobacterium tumefaciens* and select transgenic plants respectively. Further sub-culturing of the explants was conducted on M3 shooting medium to induce callus and shoot regeneration at intervals of 2–3 weeks. After 6–8 weeks of selection and when normal shoots appeared, explants were transferred to M4 rooting medium and kept at 4 °C for 2–4 weeks for vernalization. The resulting plants were placed into soil after the formation of roots. 

### 4.8. Determination of CRISPR/Cas9-Mediated Editing Events 

Genomic DNA was extracted from regenerated shoots or plants with DNA easy extraction solution [40]. Fragments covering the sgRNA target region were amplified using genotyping primers (Table S9). The PCR products from regenerated shoots or plants were sent out for sequencing (Invitrogen). Moreover, PCR products from T_2_ plants were cloned into a pMD19 T-easy cloning vector (TaKaRa). Bacterial colony PCR was conducted, and positive single colonies were chosen for sequencing. To identify editing events for the target site, the generated sequences were aligned with *BnCYP704B1a* and *BnCYP704B1b* gene sequences. The editing events were verified via checking the corresponding peaks incorporated into the sequencing reports.

### 4.9. Phenotypic Analysis

The anthers of the edited plants were collected just before anthesis. Pollen grains were released by breaking anthers and suspending them in a 1% iodine/potassium iodide solution (KI/I_2_) to assess pollen viability. The released and stained pollen was examined and photographed under a microscope (BX53, Olympus Corporation, Tokyo, Japan) as previously described [12,14]. 

## 5. Conclusions

The cytochrome P450 (CYP450) monooxygenase superfamily plays essential roles in plant growth and development via producing many primary and secondary metabolites. This study isolated a total of 687 *BnCYP450* genes in *Brassica napus* cultivar ZS11. The *BnCYP450* genes were categorized into 47 subfamilies, clustered into nine clans, and grouped into non-A-type and A-type. Gene duplication and syntenic analysis provided a clear collinear relationship between *BnCYP450s* and *AtCYP450s*. Phylogeny, gene architecture, expression profiles of the *CYP86* clan genes were determined in detail. Two *ZmMS26* orthologous genes, *BnCYP704B1a* and *BnCYP704B1b*, were observed to be specifically expressed in young anthers. Simultaneously targeted knocked-out of these two genes through CRISPR/Cas9 brought about a male-sterile line *Brassica napus*. Taken together, a global investigation of the *BnCYP450s* and targeted editing of two anther-expressed *ZmMS26* homologous genes were conducted in the current study, which could provide valuable solutions to investigate the biological significance of other *BnCYP450s* in future.

## Figures and Tables

**Table 1:** Enumeration of AtCYP450 and BnCYP450 gene superfamily.

Classifications		Family	*Arabidopsis thaliana*	*Brassica napus*	*Brassica napus* A Subgenome	*Brassica napus* C Subgenome
Different Types	Different Clans					
Non-A type	CYP51 clan	CYP51	2	9	3	6
	CYP72 clan	CYP709	3	10	4	6
		CYP714	2	5	2	3
		CYP715	1	5	2	3
		CYP72	9	25	12	13
		CYP721	1	2	1	1
		CYP734	1	2	1	1
		CYP735	2	4	2	2
	CYP710 clan	CYP710	4	6	2	4
	CYP711 clan	CYP711	1	2	1	1
	CYP74 clan	CYP74	2	4	2	2
	CYP85 clan	CYP702	6	7	3	4
		CYP707	4	17	8	9
		CYP708	4	9	6	3
		CYP716	3	0		
		CYP718	1	2	1	1
		CYP720	1	2	1	1
		CYP722	1	3	1	2
		CYP724	1	4	2	2
		CYP85	2	8	4	4
		CYP87	1	4	2	2
		CYP88	2	5	2	3
		CYP90	4	18	9	9
	CYP86 clan	CYP704	3	8	4	4
		CYP86	11	29	14	15
		CYP94	6	20	9	10
		CYP96	14	52	24	28
	CYP97 clan	CYP97	3	13	7	6
Total non-A type			95	275	129	145
A type	CYP71 clan	CYP701	1	2	1	1
		CYP703	1	4	2	2
		CYP705	25	47	19	28
		CYP706	8	16	8	8
		CYP71	50	138	72	66
		CYP712	2	4	2	2
		CYP73	1	7	3	4
		CYP75	1	2	1	1
		CYP76	9	29	12	17
		CYP77	5	16	5	11
		CYP78	6	23	11	12
		CYP79	10	25	11	14
		CYP81	18	54	23	30
		CYP82	5	13	4	9
		CYP83	2	6	3	3
		CYP84	2	9	4	5
		CYP89	7	7	4	3
		CYP93	1	2	0	2
		CYP98	3	8	4	4
Total A-type			157	412	189	222
Total			252	687	318	367

## Data Availability

Not applicable.

## Supplementary Materials

The following supporting information can be downloaded at: https://www.mdpi.com/article/10.3390/plants12020365/s1. Table S1. Details of the identified *BnCYP450* genes in *Brassica napus*; Table S2. List of tandem duplicated gene pairs of *BnCYP450s*; Table S3. List of segmental duplication of the *BnCYP450* genes across AA/CC subgenome in *Brassica napus*; Table S4. List of segmental duplication of the *BnCYP450* genes in AA subgenome; Table S5. List of segmental duplication of the *BnCYP450* genes in CC subgenome; Table S6. One-to-one orthologous relationships of the *BnCYP450* genes between *Brassica napus* and *Arabidopsis thaliana*; Table S7. Expression (TPM) of *BnCYP450* genes in different tissues; Table S8. Expression (TPM) of *BnCYP450* genes under different treatments; Table S9. Primers used in this study; Table S10. Potential off-target sites of Target-5 and Target-6 detected in GMS plants; Figure S1. Phylogenetic analysis of BnCYP450s; Figure S2. Synteny analysis of *AtCYP450* and *BnCYP450* genes; Figure S3. Alignment the protein sequences of *BnCYP704B1a* and *BnCYP704B1b*; Figure S4. Alignment the CDS sequences of *BnCYP704B1a* and *BnCYP704B1b*; Figure S5. The expression profiles of *BnCYP704B1a* and *BnCYP704B1b*; Figure S6. The sgRNA target sites and induced mutations upon *BnCYP704B1a* and *BnCYP704B1b* in regenerated shoots.
