# Supplementary material for: Genome-Wide Identification of the Cytochrome P450 Superfamily Genes and Targeted Editing of BnCYP704B1 Confers Male Sterility in Rapeseed
Source: Plants (Basel). 2023 Jan 12;12(2):365. doi: 10.3390/plants12020365 (PMC9864081; doi:10.3390/plants12020365)
Supplement: Supplementary file 1 [file plants-12-00365-s001.zip › Figure S1-S6 20230111.pdf]

Figure S1.

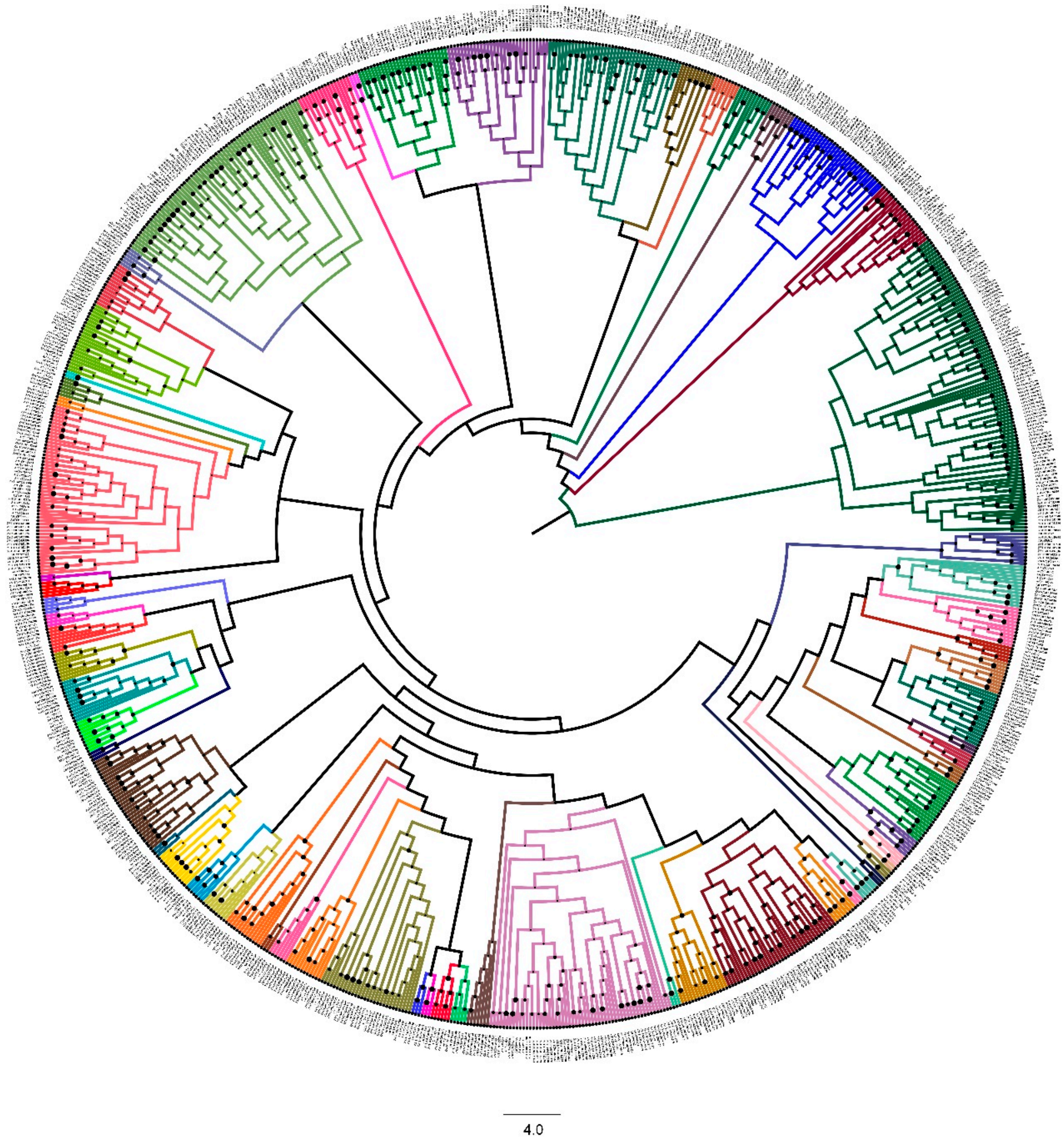

Figure S1. Phylogenetic analysis of AtCYP450s and BnCYP450s.

Figure S2.

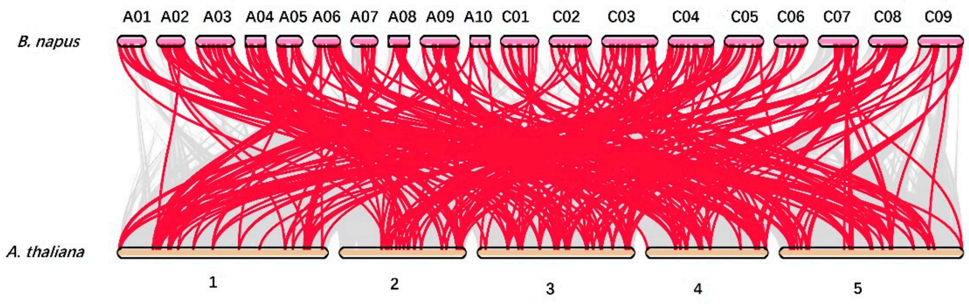

Figure S2. Synteny analysis of *AtCYP450* and *BnCYP450* genes. The gray lines indicate all collinear blocks, and the red lines highlight the orthologous relationships between *BnCYP450s* and *AtCYP450s* respectively.

Figure S3.

```
BnCYP704B1a   MSMWIVLACMLTSWIFLHRWGQRNKRGPKTWPLVGAAIEQLTNFDRMHDWLVEYLYDSRT   60
BnCYP704B1b   MSMWIVLACMVTSWIFLHRWGQRNKRGPKTWPLVGAAIEQLTNFDRMHDWLVEYLYDSRT   60
              **********:*************************************************

BnCYP704B1a   VVVPMPFTTYTYIADPINVEHVLKTNFSNYPKGETYHSYMEVLLGDGIFNSDGELWRKQR   120
BnCYP704B1b   VVVPMPFTTYTYIADPINVEHVLKTNFSNYPKGETYHSYMEVLLGDGIFNSDGELWRKQR   120
              ************************************************************

BnCYP704B1a   KTASFEFASKNLRDFSTVVFKEYSLKLFSILCQASFKDQQVDMQELLMRMTLDSICKVGF   180
BnCYP704B1b   KTASFEFASKNLRDFSTVVFKEYSLKLFSILCQASSKDQQVDMQELLMRMTLDSICKVGF   180
              *********************************** ************************

BnCYP704B1a   GVEIGTLAPDLPENRFAKAFDTANIIVTLRFIDPLWKMKKYLNIGSEALLGKSIKVVDDF   240
BnCYP704B1b   GVEIGTLAPELPENRFAKAFDTANIIVTLRFIDPLWKIKKYLNIGSEALLGKSIKVVDDF   240
              *********:**************************:***********************

BnCYP704B1a   TYSMIRRRKTEILEAQKSPSNNIKMKHDILSRFIEISDDPDSKSTEKSLRDIVLNFVIAG   300
BnCYP704B1b   TYSMIRRRKAEILEAQKSPSNNTKMKHDILSRFIEISDDPDSKSTEKSLRDIVLNFVIAG   300
              *********:************ *************************************

BnCYP704B1a   RDTTATTLTWAIYMIMMNEHVAEKLCSELQELEREKAEETNTPLRQYDTEDFKSFNERVT   360
BnCYP704B1b   RDTTATTLTWAIYMIMMNEHVAEKLCSELQELEREKAEETNTPLRQYDTEDFKSFNERVT   360
              ************************************************************

BnCYP704B1a   QFAGMLSYDSLGKLHYLHAVVTETLRLYPAVPQDPKGVLEDDILPNGTKVKAGGMVTYVP   420
BnCYP704B1b   QFAGMLSYDSLGKLHYLHAVVTETLRLYPAVPQDPKGVLEDDILPNGTKVKAGGMVTYVP   420
              ************************************************************

BnCYP704B1a   YSMGRMEYNWGSDAATFKPERWLKDGMFQNASPFKFTAFQAGPRICLGKDSAYLQMKMAM   480
BnCYP704B1b   YSMGRMEYNWGSDAAMFKPERWLKDGMFQNASPFKFTAFQAGPRICLGKDSAYLQMKMAM   480
              *************** ********************************************

BnCYP704B1a   AILCRFYKFHLVPNHPVKYRMMTILSMAHGLKVTVSRRS   519
BnCYP704B1b   AILCRFYRFHLVPNHPVKYRMMTILSMAHGLKVTVSRRS   519
              *******:*******************************
```

Figure S3. Alignment the protein sequences of *BnCYP704B1a* and *BnCYP704B1b*. The protein sequences of BnCYP704B1a and BnCYP704B1b are aligned with Clustal Omega. The 8 amino acid-replacements are indicated by yellow box.

Figure S4.

```
BnCYP704B1a   ATGTCTATGTGGATCGTTCTAGCTTGCATGCTCACATCATGGATCTTCTTGCACCGATGG   60
BnCYP704B1b   ATGTCTATGTGGATCGTTCTAGCTTGCATGGTGACATCATGGATCTTCTTGCACCGATGG   60
              ****************************** * ***************************

BnCYP704B1a   GGACAGAGGAACAAGAGAGGTCCCAAGACATGGCCTTTGGTCGGAGCAGCCATTGAGCAG   120
BnCYP704B1b   GGACAGAGGAACAAGAGAGGTCCCAAGACATGGCCTTTGGTCGGAGCAGCCATTGAGCAG   120
              ************************************************************

BnCYP704B1a   TTGACTAACTTTGACCGAATGCACGACTGGCTCGTTGAGTATCTTTACGACTCAAGAACC   180
BnCYP704B1b   TTGACTAACTTTGACCGAATGCACGACTGGCTCGTTGAGTATCTTTACGACTCGAGAACC   180
              ***************************************************** ******

BnCYP704B1a   GTAGTGGTTCCTATGCCCTTCACCACTTATACATACATAGCAGATCCCATCAATGTAGAA   240
BnCYP704B1b   GTAGTGGTTCCGATGCCCTTCACCACTTACACATACATAGCAGATCCCATCAATGTCGAA   240
              *********** ***************** ************************** ***

BnCYP704B1a   CACGTCCTCAAAACCAACTTCTCCAACTACCCAAAGGGAGAGACGTACCACTCCTATATG   300
BnCYP704B1b   CACGTCCTCAAAACCAATTTCTCCAACTACCCAAAGGGAGAGACGTACCATTCGTATATG   300
              ***************** ******************************** ** ******

BnCYP704B1a   GAAGTTTTGTTGGGAGATGGGATCTTCAATTCAGATGGAGAGCTCTGGAGGAAACAGAGG   360
BnCYP704B1b   GAAGTTTTGTTGGGAGATGGGATCTTCAATTCAGATGGAGAGCTCTGGAGGAAACAGAGG   360
              ************************************************************

BnCYP704B1a   AAAACCGCGAGTTTCGAATTTGCTTCCAAGAATCTTAGAGACTTCAGTACTGTAGTGTTT   420
BnCYP704B1b   AAAACCGCGAGTTTCGAATTTGCTTCCAAGAATCTTAGAGACTTCAGTACTGTAGTGTTT   420
              ************************************************************

BnCYP704B1a   AAAGAGTATAGCCTCAAGCTCTTCTCCATCCTTTGTCAAGCTTCTTTCAAAGACCAACAA   480
BnCYP704B1b   AAAGAGTATAGCCTCAAGCTCTTCTCCATCCTTTGTCAAGCCTCTTCCAAAGACCAACAA   480
              ***************************************** **** *************

BnCYP704B1a   GTAGACATGCAGGAACTGTTGATGAGAATGACTCTAGACTCCATATGTAAAGTGGGATTT   540
BnCYP704B1b   GTAGACATGCAGGAATTGTTGATGAGAATGACTCTAGACTCCATATGTAAAGTGGGATTT   540
              *************** ********************************************

BnCYP704B1a   GGTGTGGAGATAGGAACATTGGCTCCAGATCTACCAGAGAATCGCTTTGCTAAGGCTTTC   600
BnCYP704B1b   GGTGTGGAGATAGGAACATTAGCTCCAGAGCTACCAGAGAATCGCTTTGCTAAAGCTTTC   600
              ******************** ******** *********************** ******

BnCYP704B1a   GATACCGCAAATATAATCGTAACACTTCGTTTCATAGACCCTCTTTGGAAGATGAAAAAG   660
BnCYP704B1b   GATACCGCGAATATAATCGTAACACTTCGTTTCATAGACCCTCTTTGGAAGATTAAAAAG   660
              ******** ******************************************** ******
```

```
BnCYP704B1a   TACCTTAACATAGGATCTGAGGCATTACTTGGCAAGAGCATAAAAGTAGTCGATGATTTC   720
BnCYP704B1b   TACCTTAACATAGGATCTGAGGCATTACTTGGCAAGAGCATAAAAGTAGTCGATGATTTC   720
              ************************************************************

BnCYP704B1a   ACATATTCAATGATAAGAAGAAGGAAAACAGAGATATTAGAGGCACAAAATCTCCTTCC   780
BnCYP704B1b   ACATATTCAATGATAAGAAGAAGGAAAGCAGAGATATTAGAGGCACAGAAATCTCCTTCC   780
              *************************** ******************* ************

BnCYP704B1a   AACAACATTAAGATGAAGCATGATATACTCTCGAGGTTTATTGAGATCAGCGACGATCCT   840
BnCYP704B1b   AACAACACTAAGATGAAGCATGATATACTCTCGAGGTTCATTGAGATCAGCGACGATCCT   840
              ******* ****************************** *********************

BnCYP704B1a   GATAGCAAATCAACTGAGAAAAGCCTAAGAGATATAGTCCTCAACTTTGTTATTGCTGGA   900
BnCYP704B1b   GATAGCAAATCAACTGAGAAAAGCCTAAGAGACATAGTCCTTAACTTTGTTATTGCTGGA   900
              ******************************** ******** ******************

BnCYP704B1a   AGAGATACAACAGCAACAACTCTCACTTGGGCTATATATATGATAATGATGAATGAACAT   960
BnCYP704B1b   AGAGATACAACAGCAACAACTCTCACTTGGGCTATATACATGATAATGATGAATGAACAT   960
              ************************************** *********************

BnCYP704B1a   GTGGCCGAGAAGCTTTGCTCAGAGCTACAAGAACTCGAAAGAGAAAAGGCCGAAGAGACA   1020
BnCYP704B1b   GTCGCCGAGAAGCTTTGCTCAGAGCTACAAGAACTCGAAAGAGAAAAGGCAGAAGAGACA   1020
              ** *********************************************** *********

BnCYP704B1a   AACACACCGTTGCGTCAATACGATACAGAGGACTTCAAGTCCTTCAACGAGAGGGTAACA   1080
BnCYP704B1b   AACACACCGTTGCGTCAATACGATACAGAGGACTTCAAGTCCTTCAACGAGAGGGTAACA   1080
              ************************************************************

BnCYP704B1a   CAGTTTGCAGGAATGTTGAGTTATGATTCTTTAGGAAAATTACATTACTTACATGCTGTG   1140
BnCYP704B1b   CAGTTTGCAGGAATGTTGAGTTATGATTCTTTAGGAAAATTACACTACTTACATGCCGTG   1140
              ******************************************** *********** ***

BnCYP704B1a   GTAACAGAAACACTTCGTCTCTACCCAGCAGTTCCTCAGGATCCAAAAGGAGTGTTAGAA   1200
BnCYP704B1b   GTAACAGAAACACTTCGTCTCTACCCAGCAGTTCCTCAGGATCCAAAAGGAGTGTTAGAA   1200
              ************************************************************

BnCYP704B1a   GATGATATATTGCCTAATGGAACAAAAGTAAAAGCAGGAGGTATGGTAACATATGTTCCT   1260
BnCYP704B1b   GATGATATATTACCTAATGGAACAAAAGTAAAAGCAGGAGGAATGGTAACATATGTTCCT   1260
              *********** ***************************** ******************

BnCYP704B1a   TACTCAATGGGTCGTATGGAATACAACTGGGGATCAGATGCAGCCACGTTTAAACCCGAG   1320
BnCYP704B1b   TACTCAATGGGTCGTATGGAGTACAACTGGGGATCAGATGCAGCAATGTTTAAACCAGAA   1320
              ******************** *********************** * ********* ** 
```

```
BnCYP704B1a   AGATGGCTTAAAGATGGGATGTTTCAGAACGCTTCCCCATTCAAGTTCACAGCATTTCAG   1380
BnCYP704B1b   AGATGGCTTAAAGATGGGATGTTTCAGAACGCTTCCCCATTCAAGTTCACAGCATTTCAG   1380
              ************************************************************

BnCYP704B1a   GCTGGACCTAGGATATGCTTGGGAAAGGATTCAGCTTATCTACAAATGAAGATGGCGATG   1440
BnCYP704B1b   GCTGGACCTAGAATATGCTTGGGAAAAGATTCAGCTTATCTACAAATGAAGATGGCAATG   1440
              *********** ************** ***************************** ***

BnCYP704B1a   GCAATTCTTTGCAGATTTTATAAGTTCCATTTGGTACCAAATCATCCTGTCAAGTACCGG   1500
BnCYP704B1b   GCAATTCTTTGCAGATTCTATAGATTCCATTTGGTACCAAATCATCCTGTCAAGTACCGG   1500
              ***************** ****  ************************************

BnCYP704B1a   ATGATGACAATTCTATCTATGGCGCATGGTTTGAAAGTTACTGTATCCAGACGTTCATAG   1560
BnCYP704B1b   ATGATGACAATTCTATCTATGGCGCATGGTTTGAAAGTTACTGTATCCAGACGTTCATAG   1560
              ************************************************************
```

Figure S4. Alignment the CDS sequences of *BnCYP704B1a* and *BnCYP704B1b*. The CDS sequences of *BnCYP704B1a* and *BnCYP704B1b* are aligned with Clustal Omega. The 41 nucleotide-replacements are indicated by green box.

Figure S5.

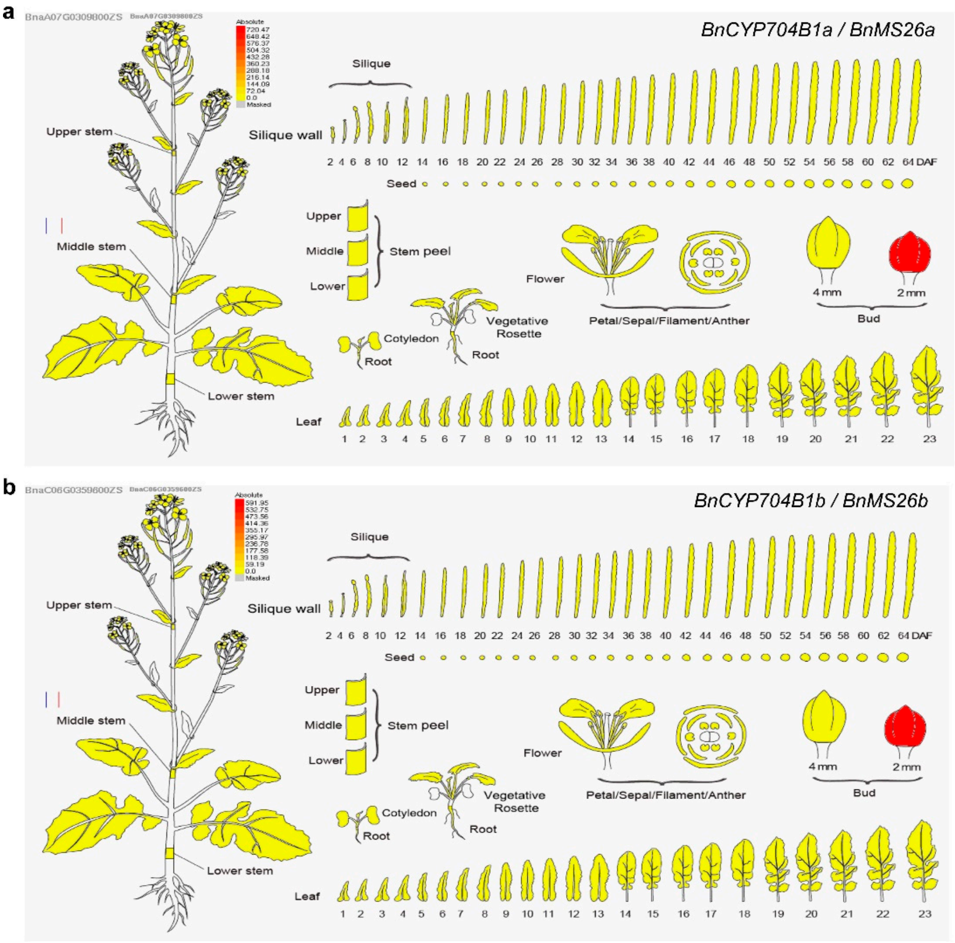

Figure S5. The expression profiles of *BnCYP704B1a* and *BnCYP704B1b*. The expression levels of *BnCYP704B1a* and *BnCYP704B1b* were obtained from the *Brassica napus* RNA-seq data (http://yanglab.hzau.edu.cn/BnTIR).

Figure S6.

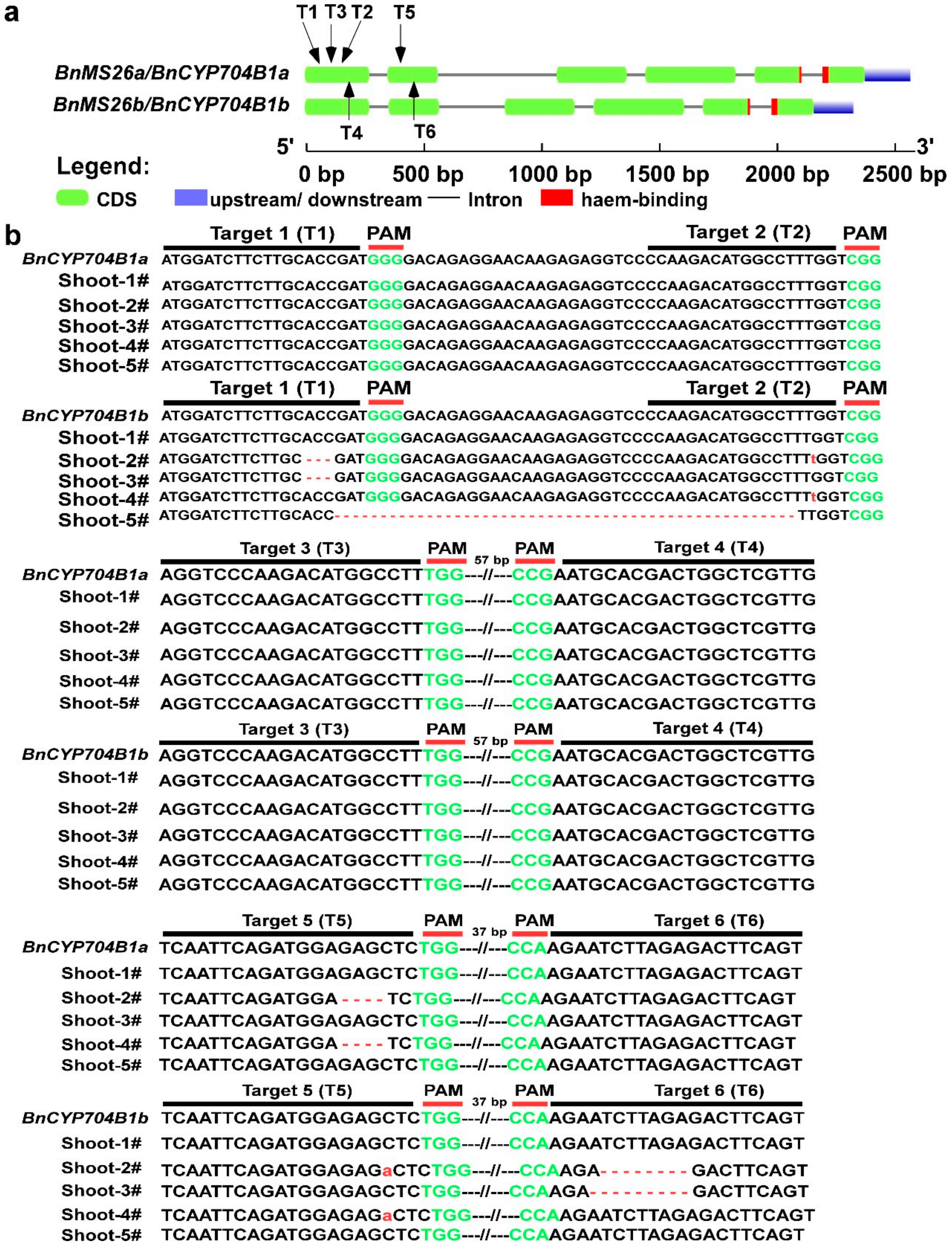

Figure S6. The sgRNA target sites and induced mutations upon *BnCYP704B1a* and *BnCYP704B1b* in regenerated shoots. (a) Gene structures of *BnCYP704B1a* and *BnCYP704B1b* and the corresponding CRISPR/Cas9 guide RNA target sites marked by black arrows. (b) Mutated genotypes of *BnCYP704B1a* and *BnCYP704B1b* in

regenerated shoots derived from infected hypocotyl explants. The target sites sequences are marked in *BnCYP704B1a* and *BnCYP704B1b* sequences with PAM green highlighted.
